# *R* 3 *c*-type LnNiO_3_ (Ln = La, Ce, Nd, Pm, Gd, Tb, Dy, Ho, Er, Lu) half-metals with multiple Dirac cones: a potential class of advanced spintronic materials

**DOI:** 10.1107/S2052252519012570

**Published:** 2019-10-16

**Authors:** Xiaotian Wang, Guangqian Ding, Zhenxiang Cheng, Hongkuan Yuan, Xiao-Lin Wang, Tie Yang, Rabah Khenata, Wenhong Wang

**Affiliations:** a Institute for Superconducting and Electronic Materials (ISEM), University of Wollongong, North Wollongong NSW 2500, Australia; b Institute for Quantum Information and Spintronics (IQIS), School of Science, Chongqing University of Posts and Telecommunications, Chongqing 400065, People’s Republic of China; c School of Physical Science and Technology, Southwest University, Chongqing 400715, People’s Republic of China; d Laboratoire de Physique Quantique de la Matière et de Modélisation Mathématique (LPQ3M), Université de Mascara, Mascara 29000, Algeria; e State Key Laboratory for Magnetism, Beijing National Laboratory for Condensed Matter Physics and Institute of Physics, Chinese Academy of Sciences, Beijing 100190, People’s Republic of China

**Keywords:** rhombohedral materials, Dirac half-metals, DHMs, electronic structures, spintronics, first-principles studies, density functional theory, materials modeling

## Abstract

In the past three years, Dirac half-metals (DHMs) have attracted considerable attention and become a high-profile topic in spintronics becuase of their excellent physical properties such as 100% spin polarization and massless Dirac fermions. Two-dimensional DHMs proposed recently have not yet been experimentally synthesized and thus remain theoretical. As a result, their characteristics cannot be experimentally confirmed. In addition, many theoretically predicted Dirac materials have only a single cone, resulting in a nonlinear electromagnetic response with insufficient intensity and inadequate transport carrier efficiency near the Fermi level. Therefore, after several attempts, we have focused on a novel class of DHMs with multiple Dirac crossings to address the above limitations. In particular, we direct our attention to three-dimensional bulk materials. In this study, the discovery via first principles of an experimentally synthesized DHM LaNiO_3_ with many Dirac cones and complete spin polarization near the Fermi level is reported. It is also shown that the crystal structures of these materials are strongly correlated with their physical properties. The results indicate that many rhombohedral materials with the general formula LnNiO_3_ (Ln = La, Ce, Nd, Pm, Gd, Tb, Dy, Ho, Er, Lu) in the space group *R* 3 *c* are potential DHMs with multiple Dirac cones.

## Abstract 2

In this study, an experimentally synthesized DHM LaNiO_3_ is discovered with many Dirac cones and complete spin polarization near the Fermi level via first principles; it is also shown that the crystal structures of these materials are strongly correlated with their physical properties.

What are the key challenges lying ahead for the next generation of spintronics (Wang *et al.*, 2016 ▸, 2018 ▸; Wang, 2017 ▸)? This question relates to a key issue encountered in the current study – how to achieve ultra-fast transmission and zero-energy dissipation. To obtain a dissipation-free spin current, we found that the key to addressing this issue is identifying new materials with linear energy band crossings (Dirac cone features) and high spin polarization near the Fermi level. For this purpose, two types of materials with high-polarization, Dirac half-metals (DHMs) (Kan *et al.*, 2011 ▸; Liu *et al.*, 2017 ▸; Li & Yang, 2017 ▸; He *et al.*, 2016 ▸) and Dirac spin-gapless semiconductors (DSGSs) (He *et al.*, 2017 ▸; Wang *et al.*, 2010 ▸; Wang, 2008 ▸), were predicted. DHMs exhibit a novel Dirac cone in one spin direction and a semiconductor/insulator property in the other; thus, in theory, the entire material has a spin polarization of 100%. Unlike non-spin-polarized Dirac structures (Neto *et al.*, 2009 ▸; Zhang *et al.*, 2017 ▸) such as graphene, DHMs can break the time reversal symmetry (TRS) in spin-resolved orbital physics because of the differences in the orders of charge current and spin current under TRS and the non-dissipative property of intrinsic coupling in DHMs. True DSGSs, which can be considered as extreme cases of DHMs, are relatively difficult to achieve. Therefore, the theoretical search for DHMs is critical for overcoming the current bottleneck in spintronics.

The concept of a DHM was first proposed in a triangular ferromagnet (Ishizuka & Motome, 2012 ▸). However, these materials are primarily limited to two-dimensional layered materials and heterojunction systems that have not yet been synthesized (Liu *et al.*, 2017 ▸) such as CrO_2_/TiO_2_ heterostructures and NiCl_3_ monolayers. Little progress was made until 2017, when Du’s team discovered a novel category of three-dimensional DHMs with multiple Dirac cones (MDCs) based on MnF_3_ (Jiao *et al.*, 2017 ▸), which have been realized experimentally. Based on first-principles calculations, Du and coworkers also investigated LaMnO_3_ (Ma *et al.*, 2018 ▸) as a novel DHM and found that the material had multiple Dirac-like half-metallic properties.

Inspired by the above work, we extensively searched bulk materials for DHMs with MDCs and found that rhombohedral structures with the space group 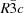 provide many potential DHMs for exploration. By comparing the DHMs identified in our search with MnF_3_, we discovered a series of materials with the general formula LnNiO_3_ that also has many Dirac cones near the Fermi level, similar to MnF_3_. Importantly, the LaNiO_3_ material with an 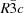 structure has already been prepared by Sreedhar *et al.* (1992 ▸).

All calculations in this manuscript were carried out in *VASP* code (Hafner, 2007 ▸) using spin-polarized density functional theory (DFT). It should be noted that the GGA-PBE (Peverati & Truhlar, 2011 ▸) method has been widely used to predict the half-metallic and spin-gapless semiconducting properties of bulk materials (Wang *et al.*, 2017*a* ▸; Qin *et al.*, 2017 ▸), and many spin-gapless semiconductors and half-metallic bulk materials predicted by GGA-PBE have been experimentally synthesized and confirmed (Ouardi *et al.*, 2013 ▸; Bainsla *et al.*,2015 ▸). More details about the methods of calculation can be found in the supporting information.

First, taking LaNiO_3_ as an example, we discuss its crystal structure and electronic structure in detail. The crystal structure of LaNiO_3_ is shown in Fig. S1 of the supporting information; a hexagonal structure with the space group 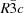 (No. 167) which contains octahedrally coordinated metal centers with equal Ni—O bond lengths of 1.954 Å. The optimized equilibrium lattice parameters are *a* = *b* = 5.499 Å and *c* = 13.078 Å. The X-ray powder diffraction patterns of LaNiO_3_ have been studied by Sreedhar *et al.* (1992 ▸) more than 20 years ago and the experimental lattice constants (*a* = *b* = 5.49 Å, *c* = 13.14 Å) of this material show a good qualitative agreement with the DFT results in the current study. The unit cell is composed of 6 La, 6 Ni and 18 O atoms. The total magnetic moment obtained through calculation was 6.236 µ_B_, and the magnetic properties were mainly contributed by Ni atoms (see Table 1 ▸). The magnetic contribution of each Ni atom (1.326 µ_B_) was higher than that of each O and La atom. To further clarify the magnetic structure of LaNiO_3_, Fig. 1 ▸ shows the ferromagnetic (FM), antiferromagnetic (AFM) and nonmagnetic (NM) states of the 1 × 1 × 1 unit cell [Figs. 1 ▸(*a*)–1(*d*)]. The total energy selected under the FM magnetic structure was 0 meV. In the 1 × 1 × 1 unit cell, two AFM states were considered simultaneously [Figs. 1 ▸(*c*) and 1(*d*)]. The calculations revealed that the NM magnetic structure was the most unstable and the total energy of the two AFM states fell between the energies of the FM and NM states. This demonstrates that the FM structure of LaNiO_3_ is most stable in the case of the 1 × 1 × 1 unit cell.

Figs. 2 ▸(*a*) and 2(*b*) show the energy band structures of LaNiO_3_ under equilibrium lattice parameters and the most stable magnetic structure. The high-symmetry points were located in the Brillouin zone, and we selected M–Γ–K–H–A–H. Normally, for some transition-metal (TM)-based materials with strongly correlated *d* electrons, the GGA+PBE method may not describe the electronic structure very well. Therefore, the GGA+*U* method (Anisimov *et al.*, 1991 ▸) should be selected in this work, namely, *U* = 6.4 eV (Balachandran *et al.*, 2018 ▸) was added to the Ni-*d* orbital during the DFT calculation. As shown in Fig. 2 ▸(*a*), LaNiO_3_ showed multiple linear energy band dispersions (Dirac cones) near the Fermi level in the spin-up direction. Specifically, one Dirac cone was located at the A high-symmetry point, four Dirac cones were distributed along the M–Γ interval, two Dirac cones were distributed along the Γ–K direction and one Dirac cone was distributed along the A–H direction. It should be noted that more Dirac cones would have been discovered if we had considered all the high-symmetry points in the Brillouin zone. In general, all of the Dirac cones in 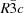-type LaNiO_3_ are protected by the *D*_3*d*_ symmetry. A detailed band symmetry analysis can be seen in Table S1 of the supporting information. Obviously, one can see that the bands near the Fermi level belong to two-dimensional irreducible representation E.

Compared with DHMs with one Dirac cone, the multiple Dirac energy band dispersions are expected to result in a stronger nonlinear electromagnetic response and a higher efficiency of carrier transport (because multiple Dirac channels exist at the Fermi level). These results of spin-up channel are similar to those reported by Du and coworkers, who also demonstrated the existence of MDCs in LaCuO_3_ (Zhang *et al.*, 2018 ▸). However, unlike the non-spin polarization system LaCuO_3_, the spin-polarized Dirac behavior in LaNiO_3_ is intrinsic, and this system can obtain magnetism without the help of experimental techniques such as applied electric field and pressure. For LaNiO_3_, the band structures of both spin channels were also calculated according to its experimental lattice constants, as shown in Fig. 2 ▸(*c*); one can see that the electronic structure results are consistent with the theoretical ones except for very small band gap differences (∼0.06 eV).

In Fig. 2 ▸(*b*), the band structures without the effect of on-site Coulomb interaction *U* have been given. Compared with GGA+*U* results, the energy levels of the MDCs in the spin-up direction rose by approximately 0.5 eV. Also, we want to point out that the Dirac cones of LaNiO_3_ are observed in both the spin-up and spin-down directions [yellow area shown in Figs. 2 ▸(*a*) and 2(*c*)]. As shown in Fig. 2 ▸(*b*), for the case of *U* = 0 eV, the energy levels of the MDCs in the spin-down direction were approximately 0.5 eV higher than those in the spin-up direction. Spin-polarized Dirac cones have more interesting properties than conventional cones (*e.g.* those in graphene) because of the high spin polarization. For the spin-degenerate Dirac cones in graphene, the Dirac state is destroyed, although artificial modification by introducing border structures, dopants, defects and adsorbed atoms can be used to obtain magnetism. To further clarify the effect of *U* on the band structures of 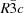 LaNiO_3_, different *U* values (*U* = 1, 3, 5 and 7 eV) were taken into consideration in these electronic structure calculations and the results are shown in Fig. S2. From this figure we can see that the MDCs in the spin-up channel are almost unchanged; however, for the half-metallic property, the effects are greater. For *U* = 0 eV and *U* = 1 eV, the electronic states and the Fermi level overlap with each other in both spin channels; therefore, the complete spin-polarization of LaNiO_3_ was lost. However, for *U* = 3, 5 and 7 eV, the 100% spin-polarization was maintained in this material. With the increase of *U* values, the band gap in the spin-down channel increases, reflecting that the half-metallic properties of this material become more and more robust.

In Fig. S3, the orbital-resolved band structure for LaNiO_3_ is plotted with the aim of furthering our understanding of the electronic structure. From it, we can see that the *p* orbitals of the O atoms and the *d* orbitals of Ni atoms mainly contribute to the total electronic structures between energies of −0.8 and 1.6 eV. In this region, the La-*d* orbital made little contribution to the band structures. That is to say, Dirac cones near the Fermi level mainly come from the hybridization between the O-*p* and Ni-*d* orbitals. We should point out that these types of MDCs arising from *d* orbitals are very rare. As shown in Fig. S4, the density-of-states results show that the states in the range −2–0 eV mainly arise from the O-*p* orbitals, and the states in the range 0–2 eV are derived from the hybridization between the O-*p* and Ni-*d* orbitals. The states ranging from 2 to 4 eV in the spin-down channel were contributed by the Ni-*d* orbital. The spin polarization (*P*) of LaNiO_3_ around the Fermi level can be obtained according to the formula
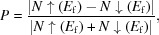
where 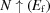 and 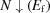 are the number of spin-up and spin down states, respectively. As shown in Fig. S4, one can see the *P* of this material is 100%, indicating LaNiO_3_ could be useful for spin injection (He *et al.*, 2019 ▸).

Fig. 3 ▸ presents a structural diagram of the energy bands, showing the effects of spin-orbit coupling (SOC) on the Dirac cones. Dirac cones still present under the influence of SOC, and the conduction and valence bands are still degenerate with respect to each other. That is to say, for the SOC effect, the MDCs near the Fermi level show strong resistance, reflecting that this material has long spin coherence which is favorable for spin transport. As shown in Fig. S5, the effects of uniform strain have been taken into consideration because the experimental lattice constants of materials always deviate from the ideal calculated equilibrium lattice constants; −8 GPa represents the compression stress of the 8 GPa imposed along the *x*, *y* and *z* directions. In contrast, the positive value represents the imposed tensile stress, from which, one can see this effect will have almost no impact on Dirac cones. The materials with robust MDCs are able to withstand external factors more easily.

In this work, we also selected LaNiO_3_ as an example to study thermodynamic properties including the thermal expansivity α, the heat capacity *C*_V_, the Gruneisen constant γ and the Debye temperature Θ_D_. Such a study was necessary. From this investigation, we were able to figure out the special properties of LaNiO_3_ under high pressures and high temperatures. The results are shown in Figs. S6–S10 and more details can be found in the supporting information.

Then, we examined the thermal stability of LaNiO_3_ at room temperature. To achieve this goal, *ab initio* molecular dynamics simulations (AIMD) were performed and a 2 × 2 × 1 superstructure of LaNiO_3_ was built. As shown in Fig. 4 ▸(*c*), this 2 × 2 × 1 superstructure contains 120 atoms (*i.e.* 24 La atoms, 24 Ni atoms and 72 O atoms) and the experiment was performed using a Nosé–Hoover thermostat at 300 K. Fig. 4 ▸(*a*) presents the fluctuations of the potential energy as a function of the simulation time (*i.e.* 2 ps) at 300 K. After 2 ps with a time step of 1 fs, we found no structural destruction of LaNiO_3_, except for some thermal-induced fluctuations [see Fig. 4 ▸(*d*)], indicating that this material is thermally stable at room temperature. Also, as shown in Fig. 4 ▸(*b*), the magnetic moment of this superstructure keeps a fixed value of 24 µ_B_, showing that the total *M*_t_ can survive at room temperature.

The electronic structures of La_0.833_NiO_3_ and LaNi_0.833_O_3_ were shown in Figs. S11(*a*) and S11(*b*), respectively. From these figures, we can further confirm that the Ni-*d* orbital contributes more to the Dirac cones near the Fermi level than La-*p* orbital. For La_0.833_NiO_3_, the Dirac cones in the spin-up channel are still recognizable even if one La atom is lost. However, for the case of LaNi_0.833_O_3_, the Dirac cones in the spin-up channel are badly damaged when one Ni atom is missing. Moreover, we should point out that, for La_0.833_NiO_3_, complete spin-polarization of system has been broken.

Finally, the crystal structure of a material is strongly correlated with its physical properties. As demonstrated in our previous study (Wang *et al.*, 2017*b* ▸), Ti-based Heusler alloys include many Hg_2_CuTi-structured materials with high spin polarization. In contrast, the spin polarization of materials with the corresponding Cu_2_MnAl structure is much lower. That is to say, this specific space group allows for the three-dimensional Dirac point to be used as symmetric protection for degeneracy. Therefore, using a series of examples, we attempted to show that rhombohedral structures with the space group 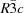 comprise an important system that includes many DHMs with MDCs with strong potential for theoretical prediction and experimental synthesis. The structural diagrams of the energy bands of a series of materials with the general formula LnNiO_3_ (Ln = Ce, Nd, Pm, Gd, Tb, Dy, Ho, Er, Lu) and space group 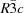 are plotted in Fig. S12. From this figure, we can see that they are all DHMs with MDCs and 100% spin-polarization. The total and atomic magnetic moments as well as the obtained equilibrium lattice constants are given in Table 1 ▸. We should point out here that the magnetic ordering of rare-earth atom Ln only occurs at very low temperature (Muñoz *et al.*, 2009 ▸; Fernández-Díaz *et al.*, 2001 ▸) and therefore, in this work, we neglect the magnetic ordering of the Ln, namely, the *f* electrons of the Ln atom are frozen into the core and the corresponding *p* and *s* states are included as valence electrons. For these LnNiO_3_ (Ln = Ce, Nd, Pm, Gd, Tb, Dy, Ho, Er, Lu) materials, many methods can be used to prepare them. For example, (i) we can synthesize bulk by solid state reaction at elevated temperatures and high oxygen pressure; (ii) we can try to prepare thin films by PLD or sol-gel methods; (iii) we can try to synthesize nano-materials by hydro­thermal methods. Hence, we hope that our current work can give some inspiration to the subsequent experimental and theoretical work, and that more 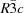-based Dirac materials will receive attention.

In summary, a series of 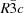-based DHMs with MDCs has been theoretically predicted: (1) LaNiO_3_, which was synthesized more than 20 years ago; and (2) LnNiO_3_ (Ln = Ce, Nd, Pm, Gd, Tb, Dy, Ho, Er, Lu), which have been predicted in terms of theory and need attention in experiment. Moreover, for experimentally synthesized material LaNiO_3_, we perform an all-round first-principle study on its electronic, magnetism and thermodynamic properties. The effects of *U*, uniform stain, SOC, vacancies and thermal stability have also been investigated via first principles, AIMD and quasi-harmonic Debye approximation. Considering the results of both Du *et al.* (Jiao *et al.*, 2017 ▸) and this study, we believe that many materials in the space group 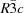 are worthy of theoretical development and experimental preparation. We hope that this letter stimulates theoretical research on DHMs with MDCs in the space group 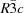. Furthermore, we believe that experimental preparation and the confirmation of predicted physical properties are imminent. This letter provides a theoretical basis for such experimental studies and is expected to help realize DHMs with MDCs in future spintronic applications.

In the supporting information we provide extensive details for the computational methods as well as the quasi-harmonic Debye model calculations, analysis of the thermodynamic properties and figures.

## Supplementary Material

Supporting information file. DOI: 10.1107/S2052252519012570/ct5010sup1.pdf

## Figures and Tables

**Figure 1:**
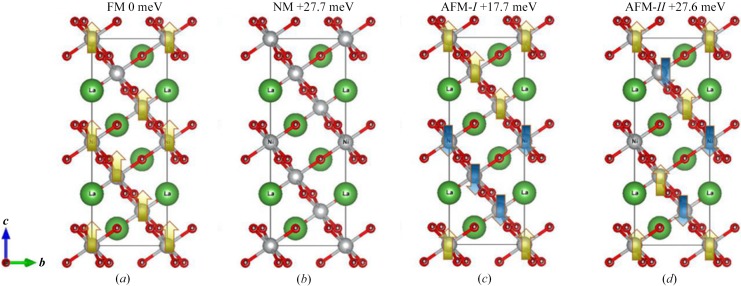
Crystal structures of LaNiO_3_; the different magnetic structures including (*a*) FM, (*b*) NM, (*c*) AFM-*I* and (*d*) AFM-*II* are taken into consideration.

**Figure 2:**
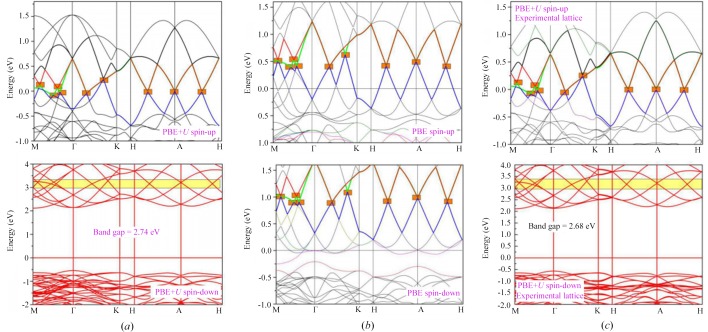
Calculated band structures of LaNiO_3_ using (*a*) GGA+*U* and (*b*) GGA methods at its optimized equilibrium lattice constants. For (*c*), the experimental lattice constants are selected during the electronic structure calculations.

**Figure 3:**
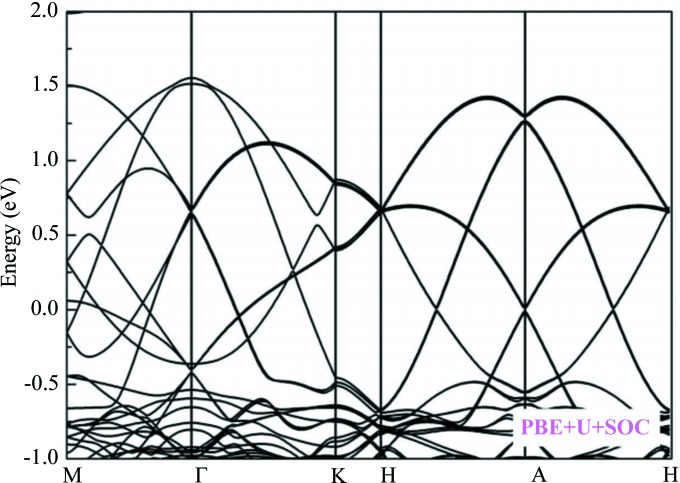
Calculated band structures of LaNiO_3_ obtained using the GGA+*U* and SOC methods at its optimized equilibrium lattice constants.

**Figure 4:**
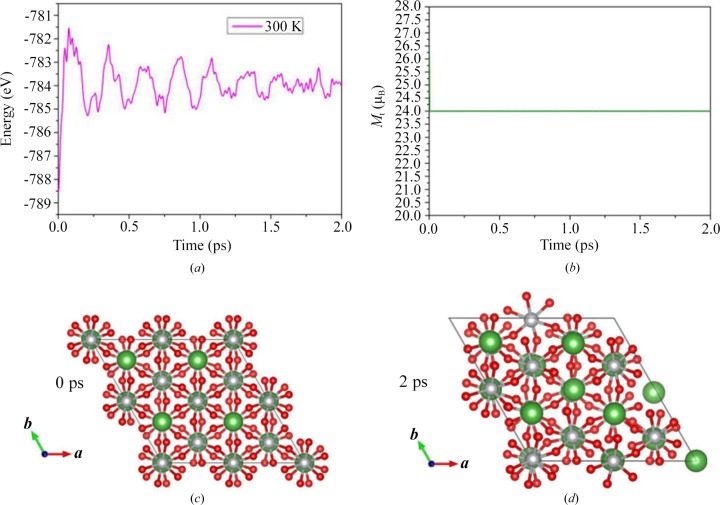
(*a*) Calculated total energy fluctuation of the superstructure and (*b*) the total magnetic moments of the superstructure for LaNiO_3_ during AIMD simulations at 300 K. The superstructures show a snapshot at the end of the simulation of (*c*) 0 ps and (*d*) 2 ps.

**Table 1:** Optimized equilibrium lattice constants of LnNiO_3_ obtained using the GGA+*U* method and their total and atomic magnetic moments at their optimized equilibrium lattice constants

LnNiO_3_	*a* (Å)	*c* (Å)	*M*_total_ (μ_B_)	*M*_Ln_ (μ_B_)	*M*_Ni_ (μ_B_)	*M*_O_ (μ_B_)
LaNiO_3_	5.499	13.078†	6.236	0.008	1.326	−0.093 /−0.103/−0.093
	5.49	13.14‡				
CeNiO_3_	5.538	13.127	6.250	0.008	1.344	−0.098/−0.109/−0.098
DyNiO_3_	5.435	12.648	6.263	0.016	1.308	−0.094/−0.095/−0.094
ErNiO_3_	5.410	12.612	6.263	0.017	1.300	−0.089/−0.092/−0.091
GdNiO_3_	5.439	13.068	6.298	0.015	1.358	−0.105/−0.108/−0.110
HoNiO_3_	5.411	12.378	6.323	0.017	1.373	−0.110/−0.114/−0.112
LuNiO_3_	5.499	13.078	6.341	0.017	1.395	−0.117/−0.120/−0.119
NdNiO_3_	5.511	12.868	6.244	0.010	1.328	−0.096/−0.102/−0.099
PmNiO_3_	5.449	13.058	6.262	0.011	1.341	−0.103/−0.107/−0.099
TbNiO_3_	5.447	12.671	6.261	0.015	1.311	−0.092/−0.096/−0.094

† From our work.

‡ Experimental parameters (Sreedhar *et al.*, 1992 ▸).
