# Supplementary material for: R 3 c-type LnNiO3 (Ln = La, Ce, Nd, Pm, Gd, Tb, Dy, Ho, Er, Lu) half-metals with multiple Dirac cones: a potential class of advanced spintronic materials
Source: IUCrJ. 2019 Oct 16;6(Pt 6):990–5. doi: 10.1107/S2052252519012570 (PMC6830210; doi:10.1107/S2052252519012570)
Supplement: Supplementary file 1 [file m-06-00990-sup1.pdf]

IUCrJ

Volume 75 (2019)

Supporting information for article:

R−3*c*-type half-metals of LnNiO3 (Ln = La, Ce, Nd, Pm, Gd, Tb, Dy, Ho, Er, Lu) with multiple dirac cones: a potential class of advanced spintronic materials

Xiaotian Wang, Guangqian Ding, Zhengxiang Cheng, Hongkuan Yuan, Xiao-Lin Wang, Tie Yang, Rabah Khenata and Wenhong Wang

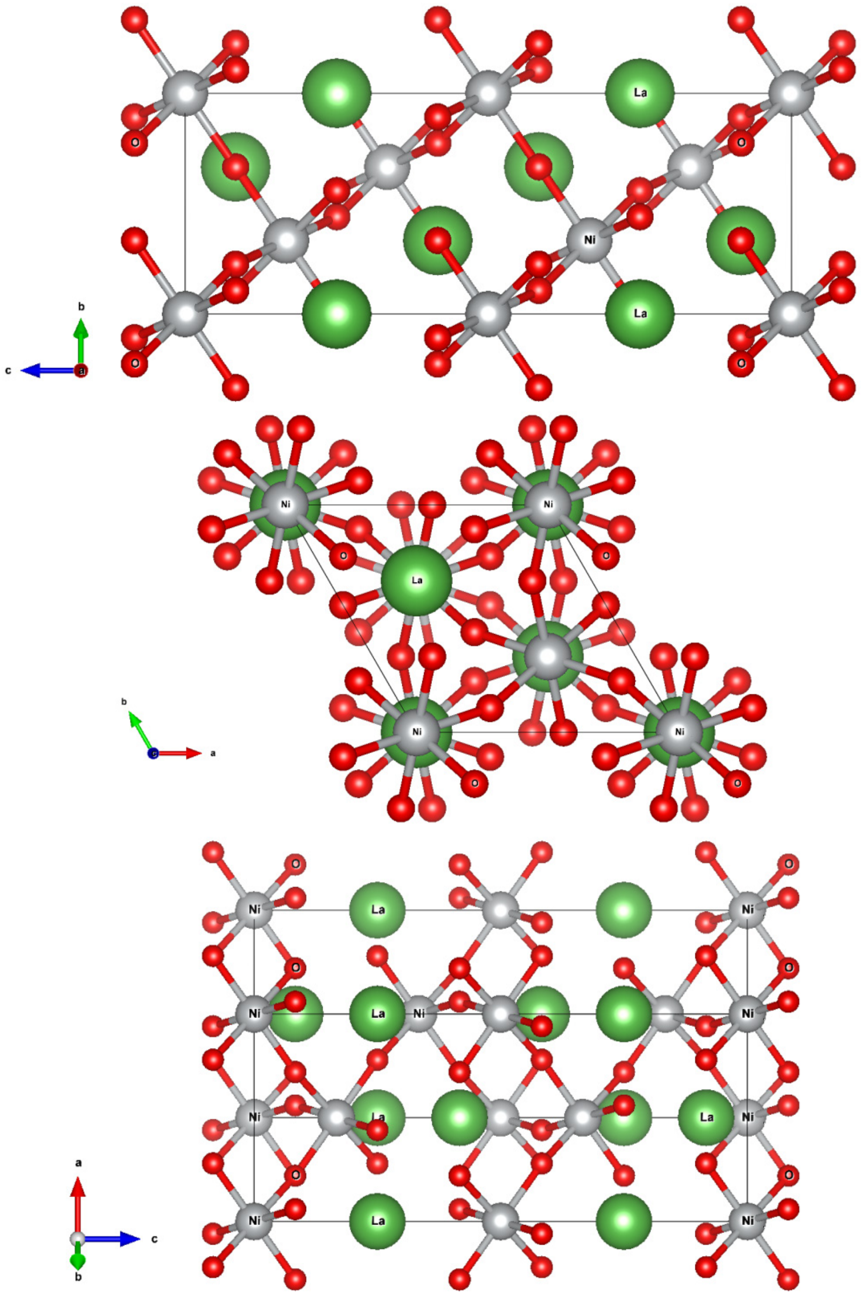

Figure S1. Crystal structures of $LaNiO_3$ material in space group $R\bar{3}c$.

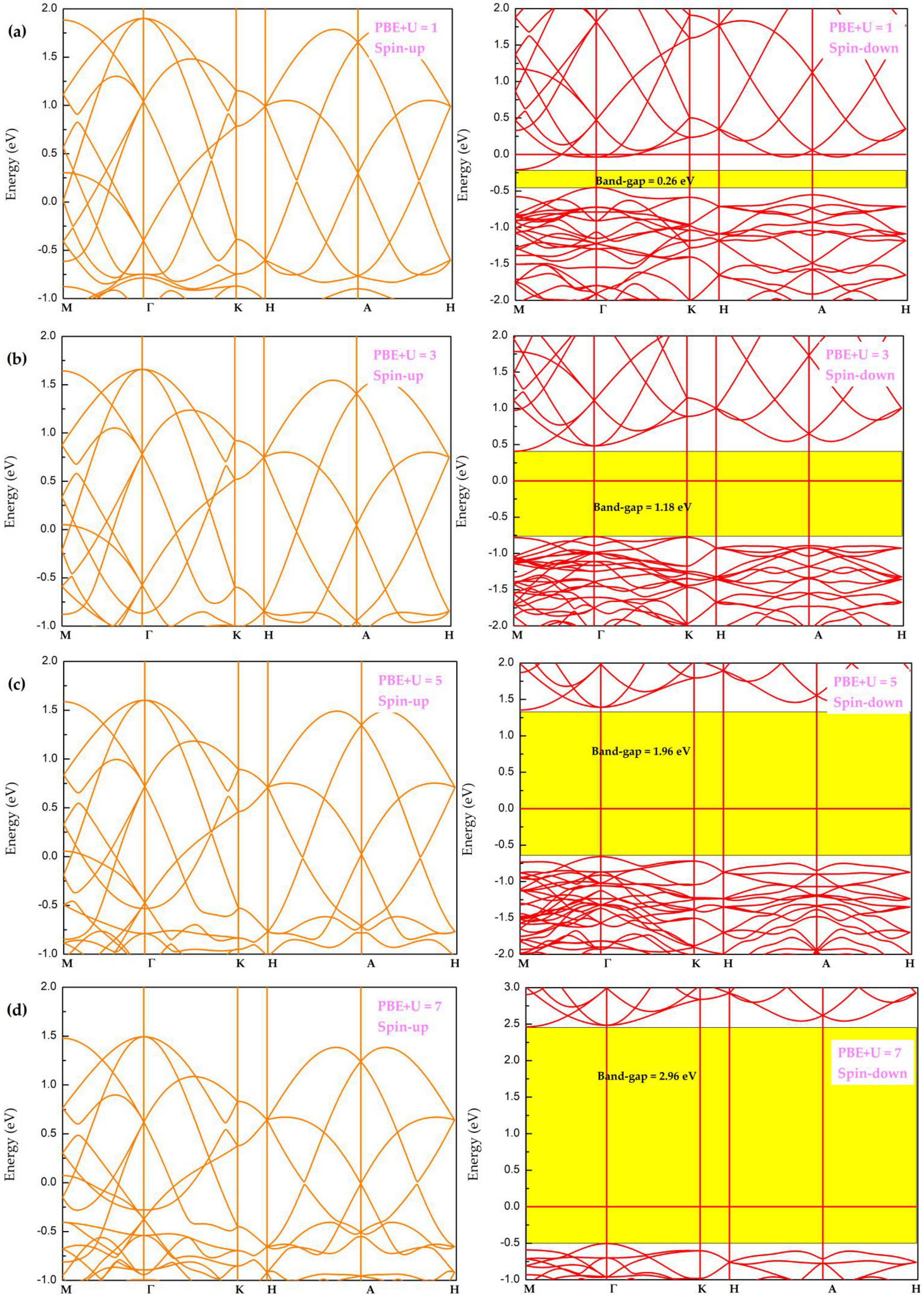

Figure S2 The calculated band structures of $LaNiO_3$ material under different U values.

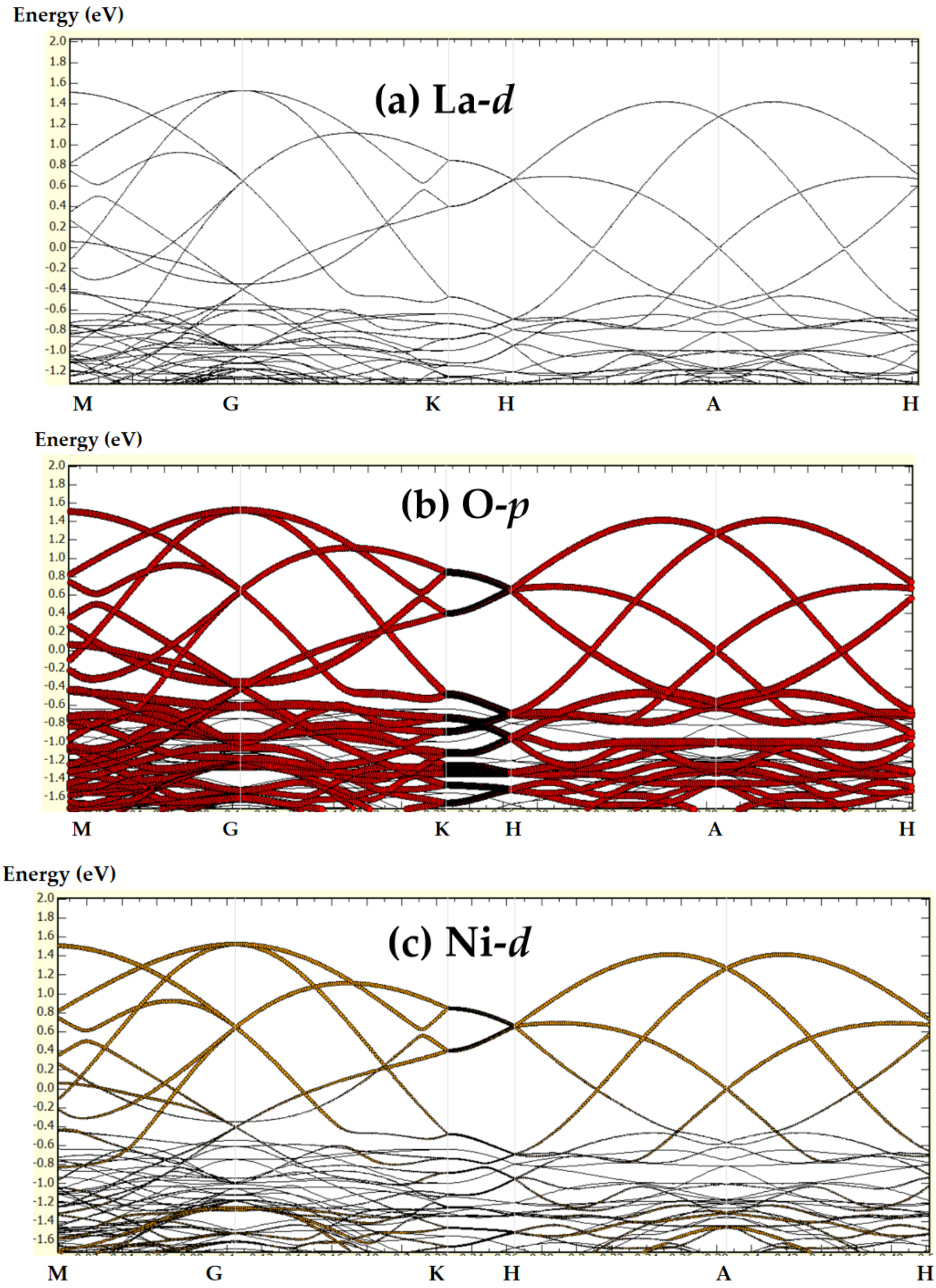

Figure S3 Orbital-resolved band structures for $LaNiO_3$ contributed by the O-p, La-d, and Ni-d orbits. The thicker line means a higher contribution, while the thinner line indicates a lower contribution.

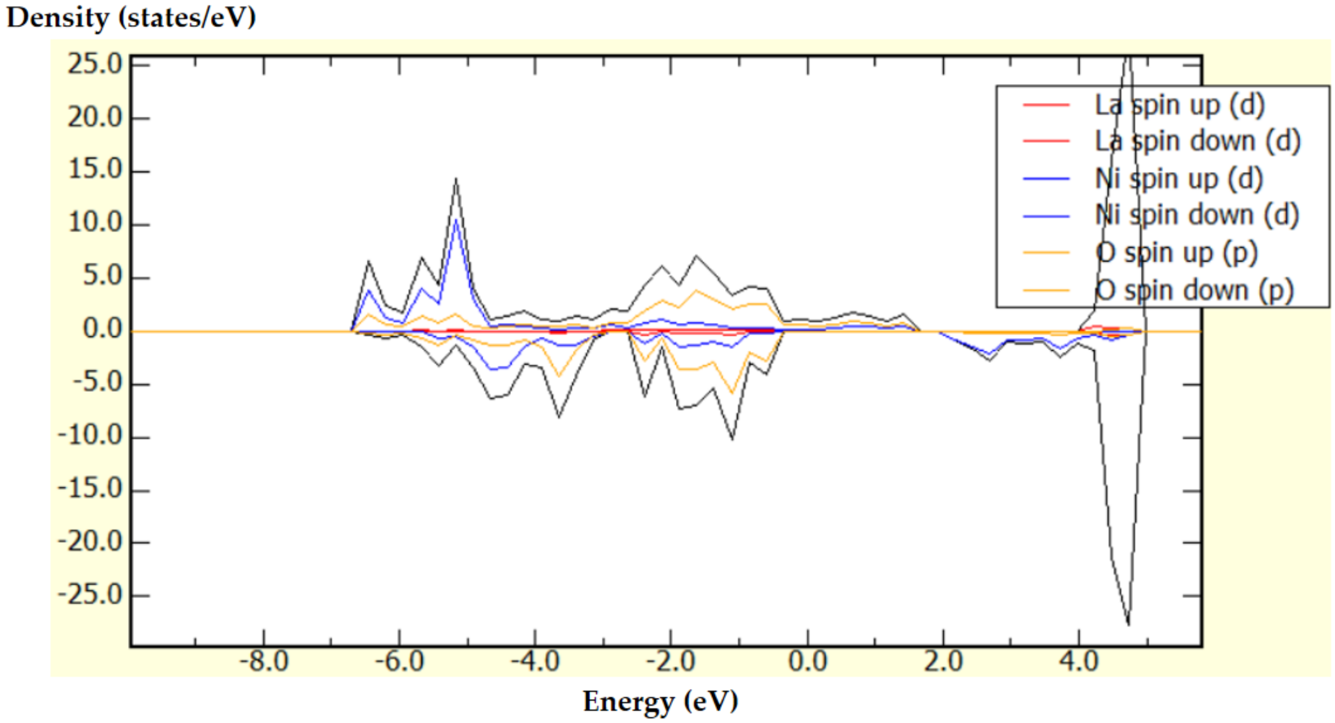

Figure S4. Calculated total and atomic DOSs for $LaNiO_3$.

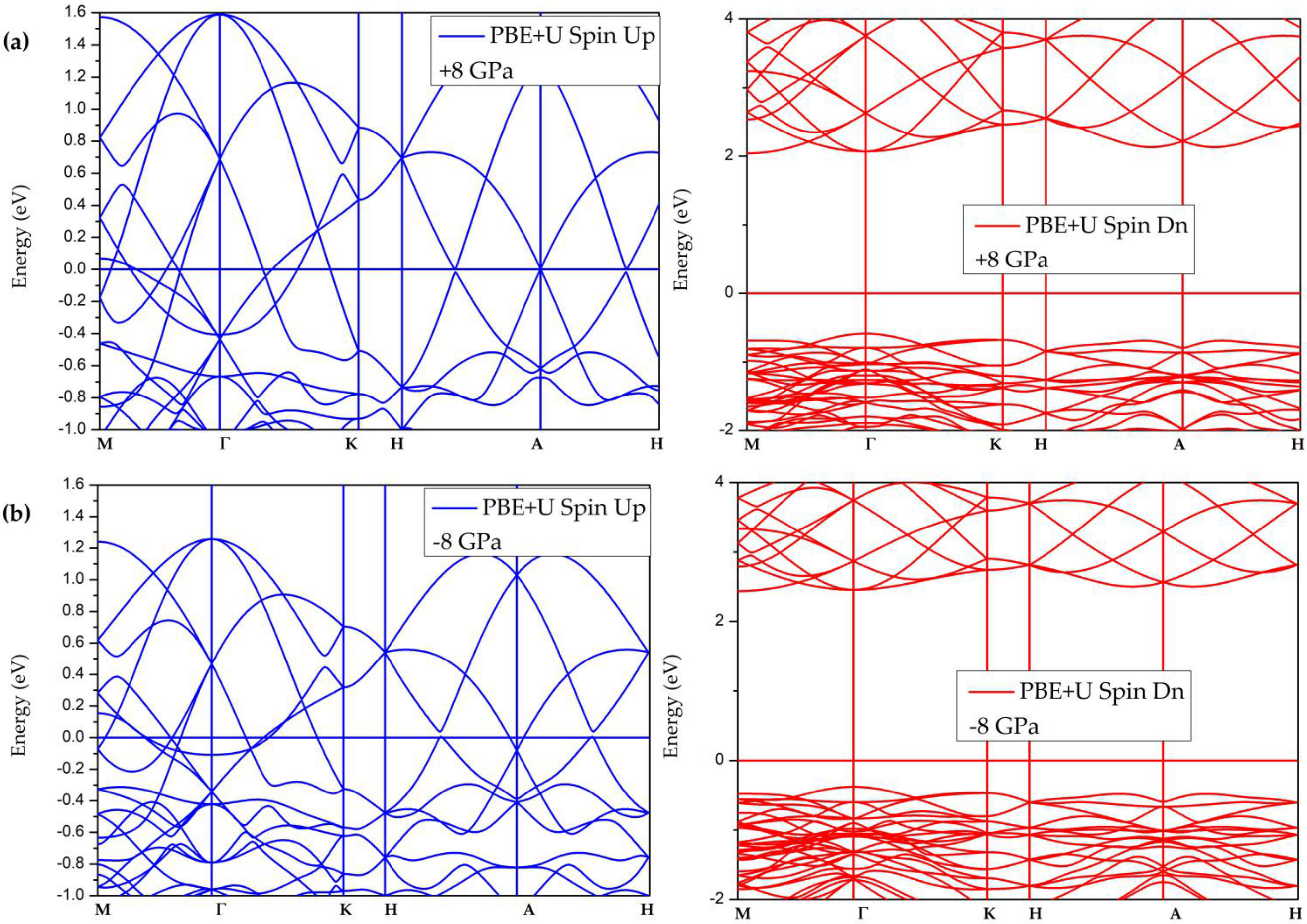

Figure S5. Calculated band structures for $LaNiO_3$ under +8 GPa and -8GPa.

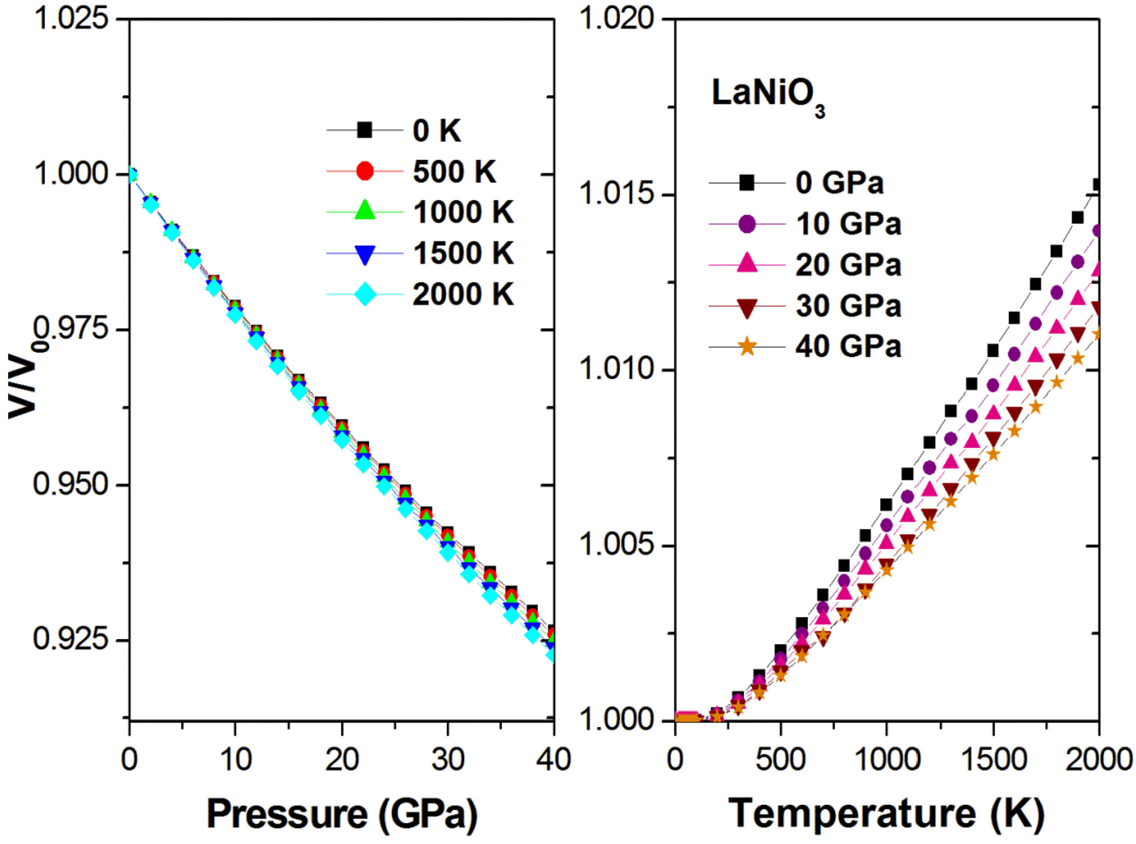

Figure S6 The normalized volume V/V_0 versus pressure and temperature for $LaNiO_3$.

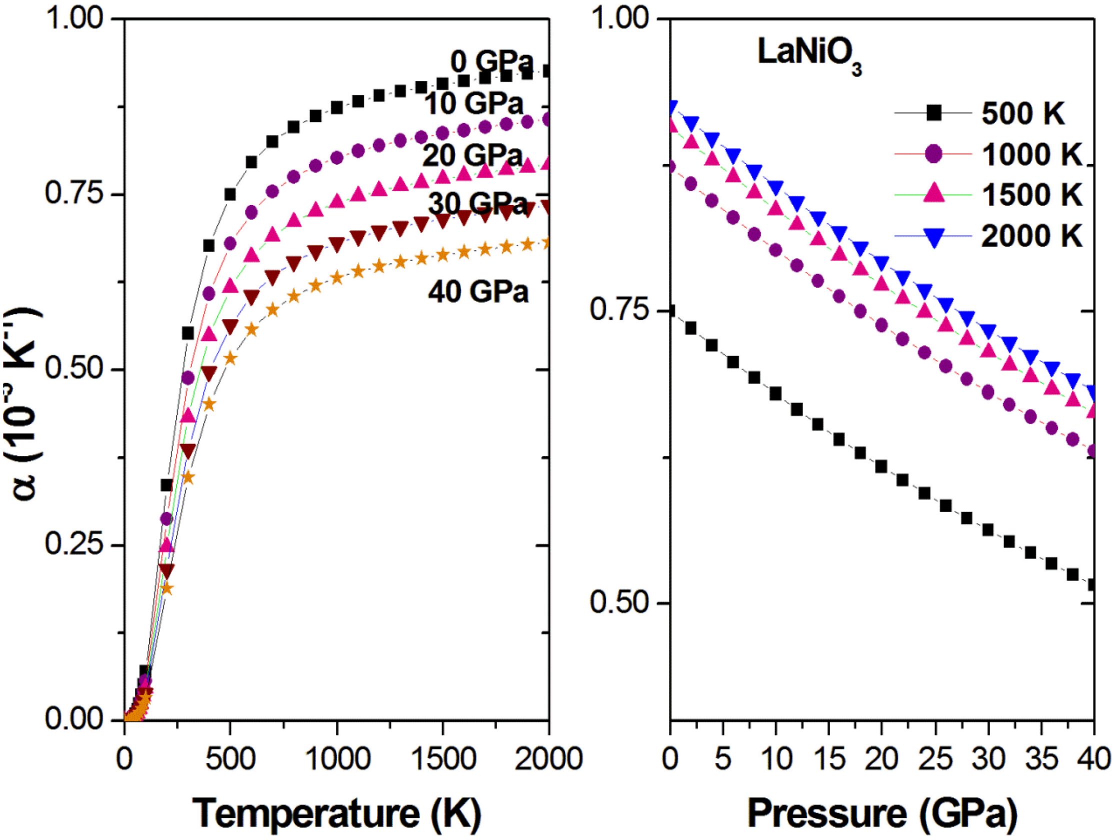

Figure S7. The thermal expansivity α versus pressure and temperature for $LaNiO_3$.

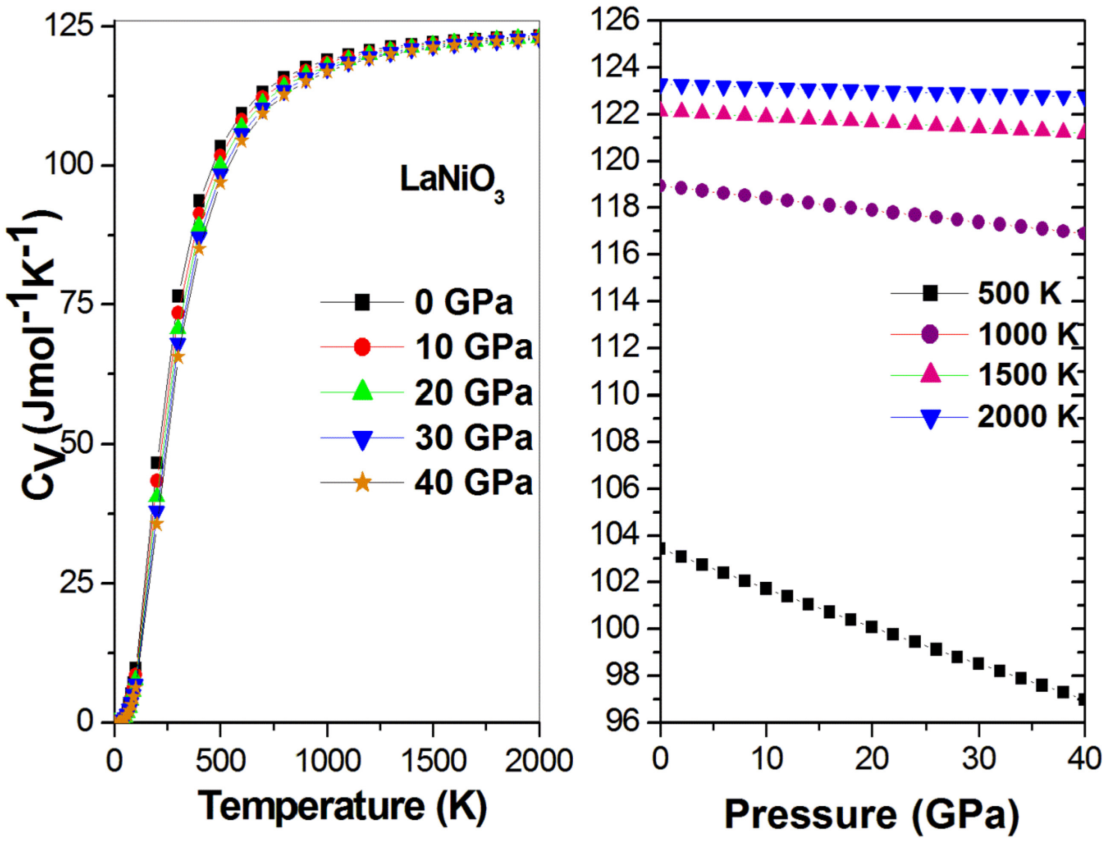

Figure S8 The heat capacity C_V versus temperature and pressure for $LaNiO_3$.

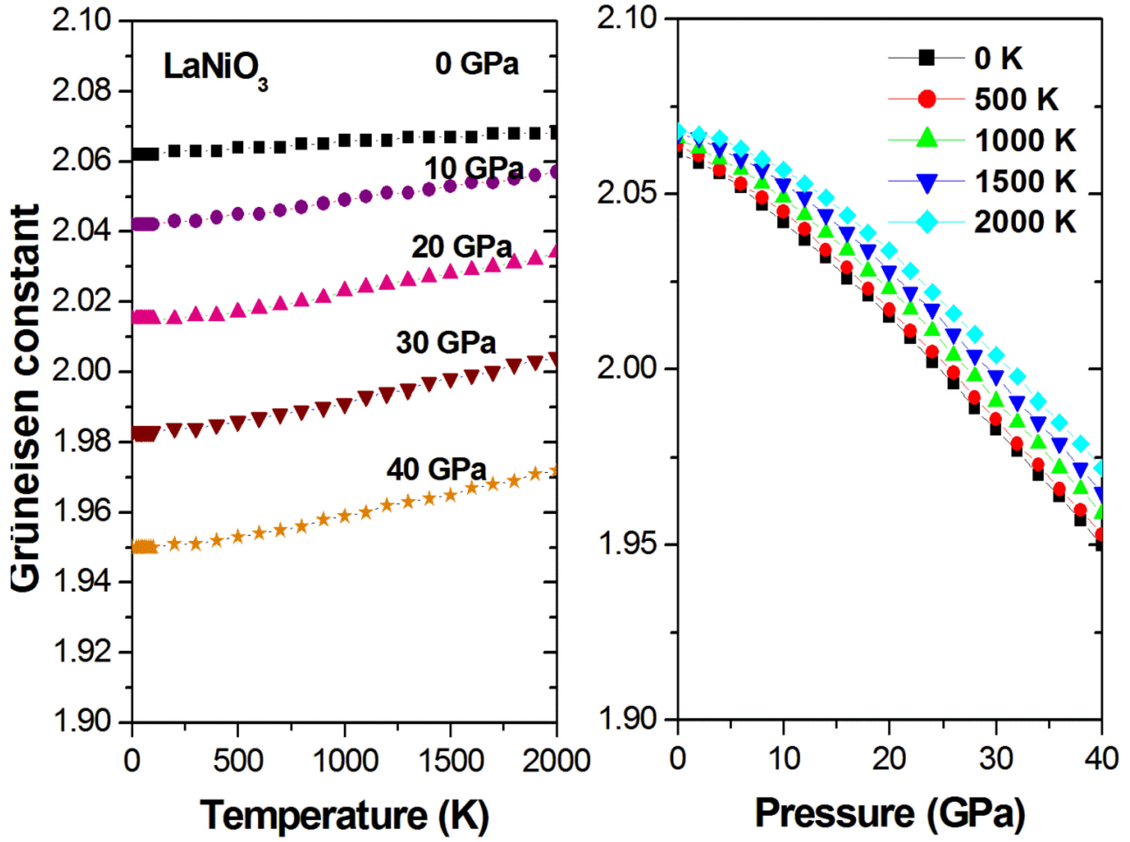

Figure S9 The Grüneisen constant γ versus pressure and temperature for $LaNiO_3$.

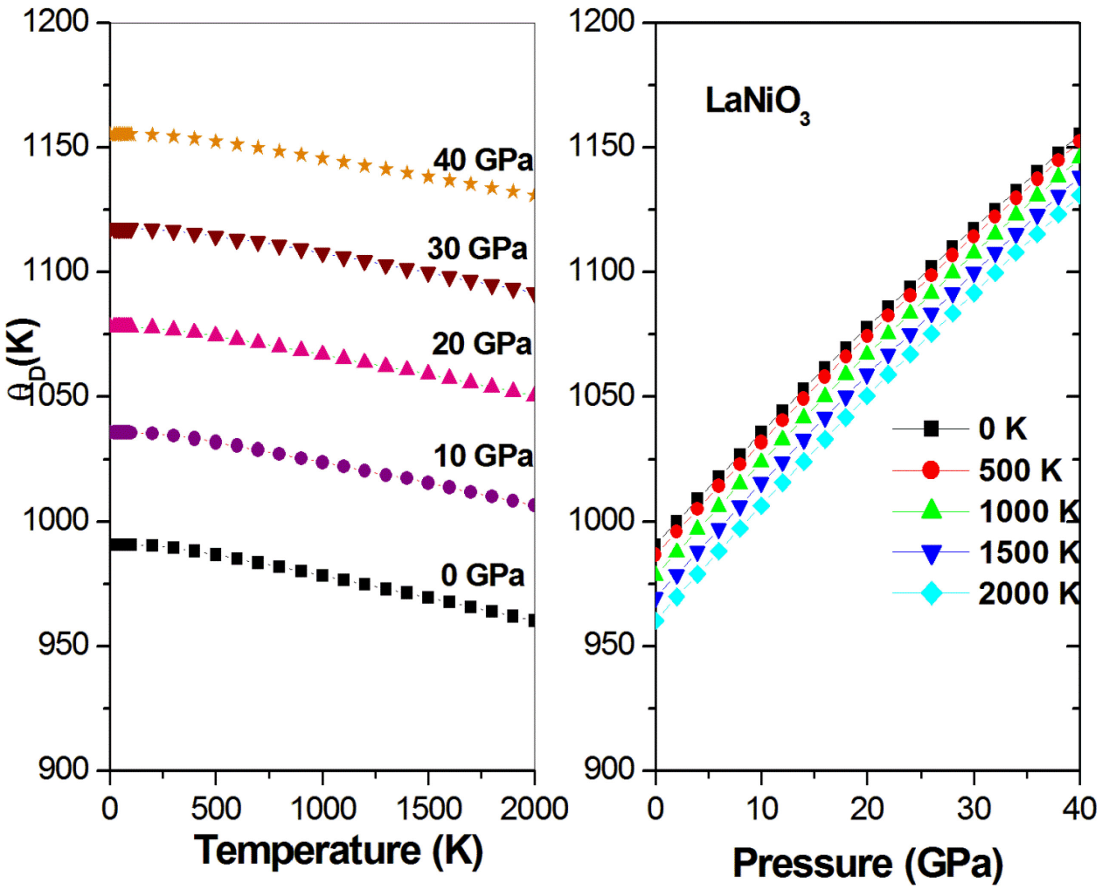

Figure S10 The Debye temperature Θ_D versus pressure and temperature for $LaNiO_3$.

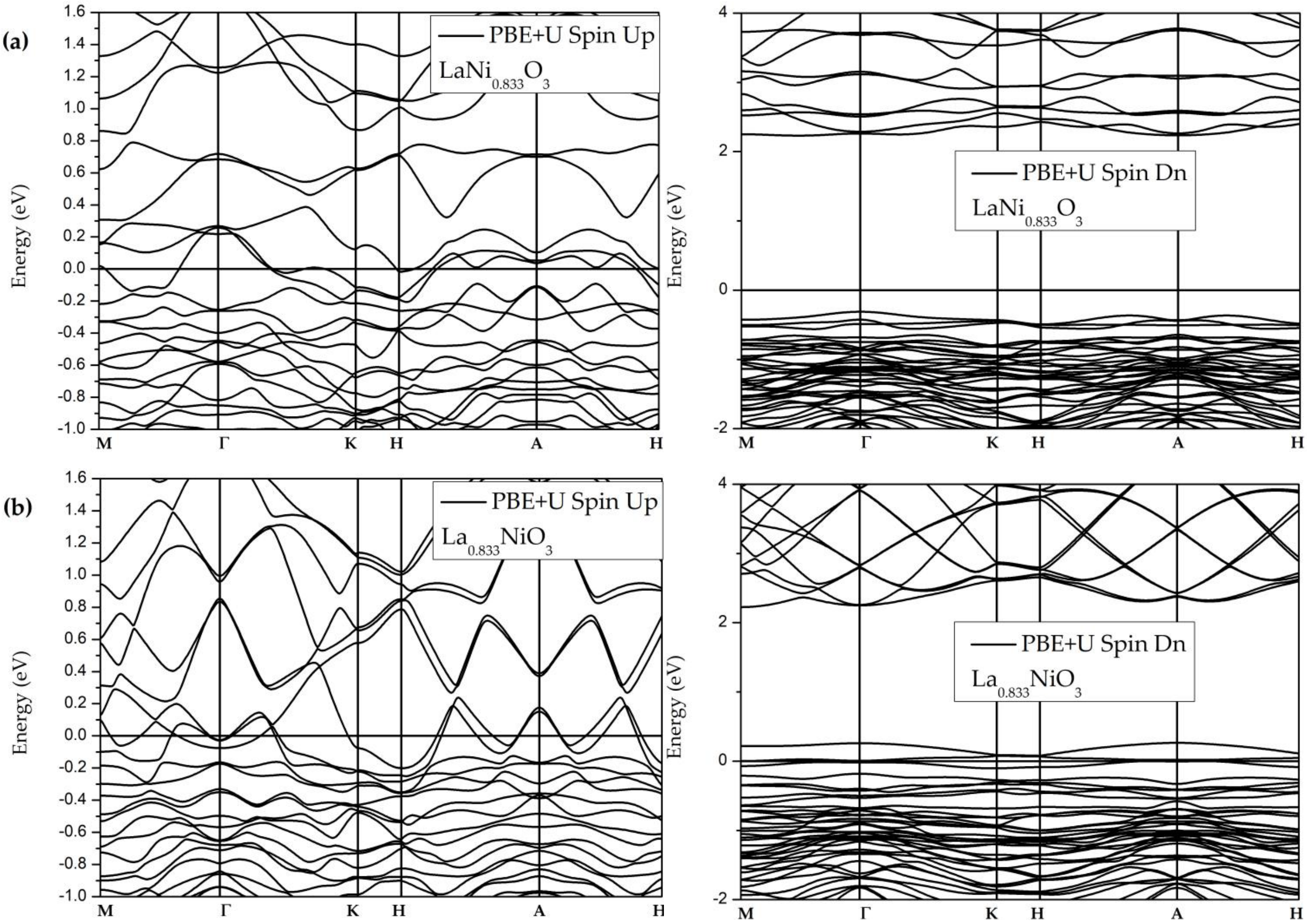

Figure S11. Calculated band structures of $La_{0.833}NiO_3$ and $LaNi_{0.833}O_3$ in both spin channels.

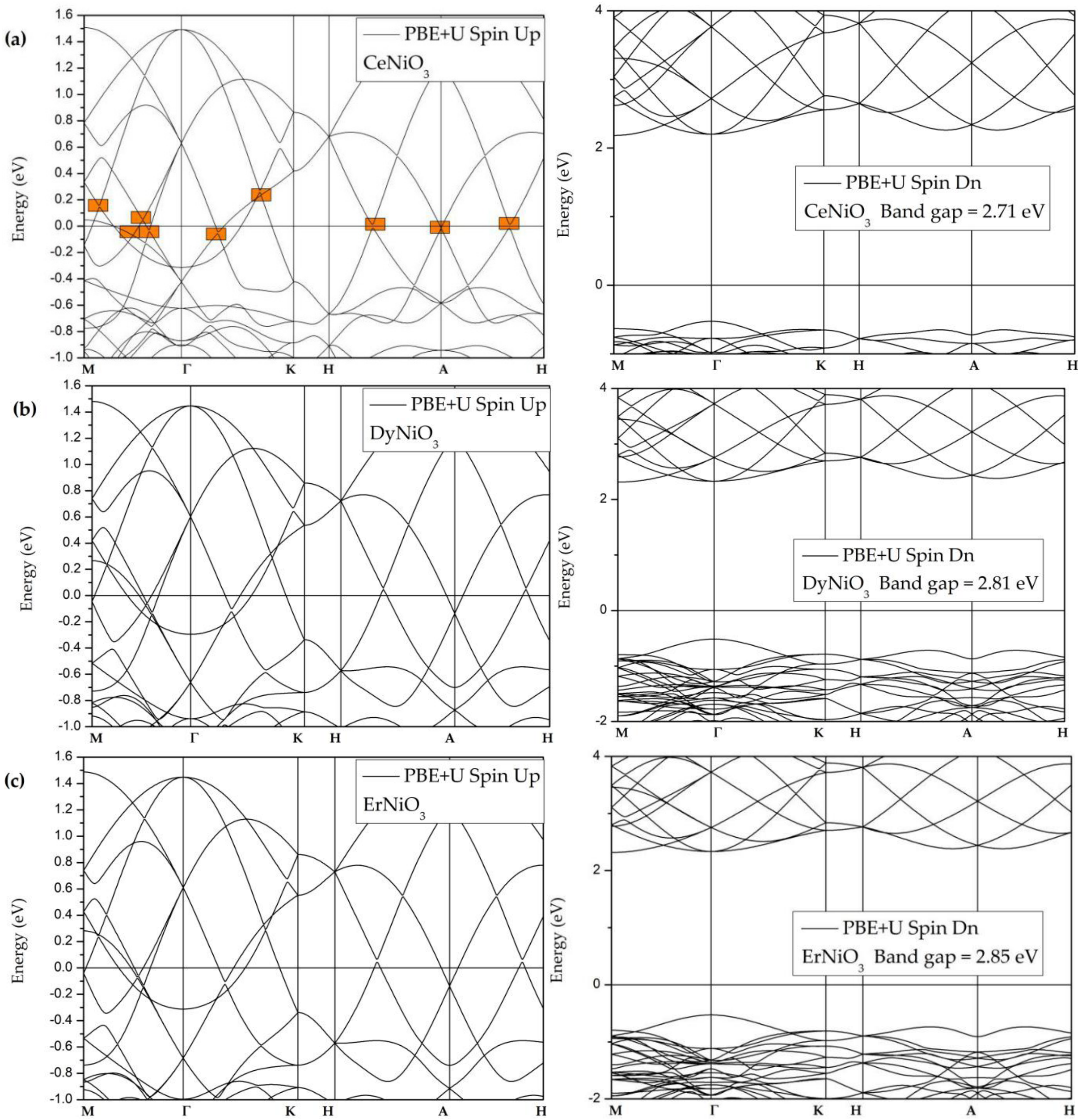

Figure S12. Cont.

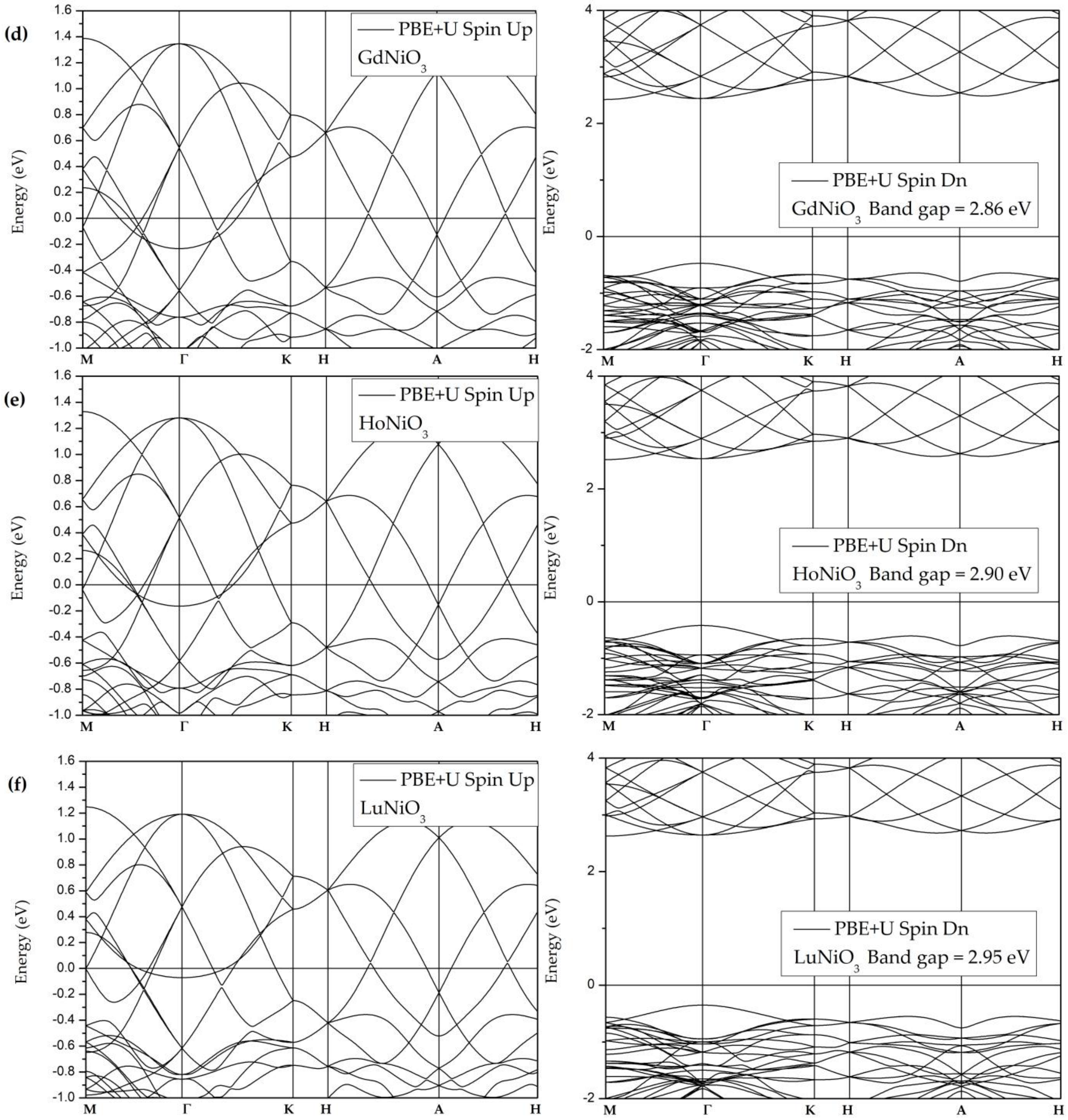

Figure S12. Cont.

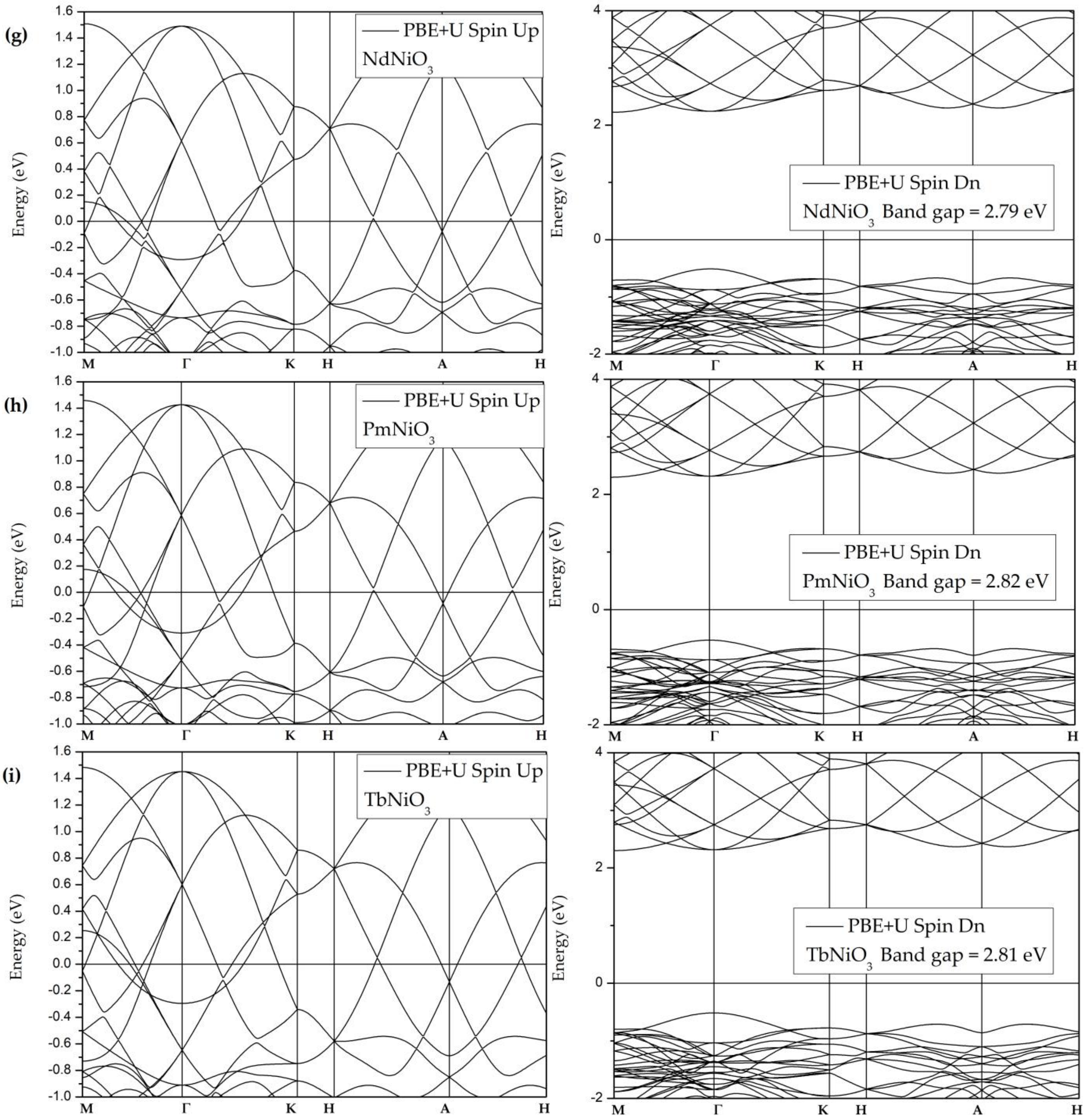

Figure S12. Calculated band structures of a series of $R\bar{3}c$ *-based DHMs with MDRs LnNiO*$_3$ *(Ln = Ce, Nd, Pm, Gd, Tb, Dy, Ho, Er, Lu).*

Table S1. Characters table for irreducible representations of the D_{3d} point group.

	E	$2C_3$	$3C_2^{'}$	i	$2S_6$	$3\sigma_d$
A_{1g}	1	1	1	1	1	1
A_{2g}	1	1	-1	1	1	-1
E_g	2	-1	0	2	-1	0
A_{1u}	1	1	1	-1	-1	-1
A_{2u}	1	1	-1	-1	-1	1
E_u	2	-1	0	-2	1	0
